# Expression Landscape of circRNAs in *Arabidopsis thaliana* Seedlings and Adult Tissues

**DOI:** 10.3389/fpls.2020.576581

**Published:** 2020-09-10

**Authors:** Anna Philips, Katarzyna Nowis, Michal Stelmaszczuk, Paulina Jackowiak, Jan Podkowiński, Luiza Handschuh, Marek Figlerowicz

**Affiliations:** ^1^ Institute of Bioorganic Chemistry, Polish Academy of Sciences, Poznan, Poland; ^2^ Institute of Computing Science, Poznan University of Technology, Poznan, Poland

**Keywords:** circRNA, *Arabidopsis thaliana*, organ-specific, RNA-seq, droplet digital PCR, data normalization

## Abstract

RNA-seq is currently the only method that can provide a comprehensive landscape of circular RNA (circRNAs) in the whole organism and its particular organs. Recent years have brought an increasing number of RNA-seq-based reports on plant circRNAs. Notably, the picture they revealed is questionable and depends on the applied circRNA identification and quantification techniques. In consequence, little is known about the biogenesis and functions of circRNAs in plants. In this work, we tested two experimental and six bioinformatics procedures of circRNA analysis to determine the optimal approach for studying the profiles of circRNAs in *Arabidopsis thaliana*. Then using the optimized strategy, we determined the accumulation of circular and corresponding linear transcripts in plant seedlings and organs. We observed that only a small fraction of circRNAs was reproducibly generated. Among them, two groups of circRNAs were discovered: ubiquitous and organ-specific. The highest number of circRNAs with significantly increased accumulation in comparison to other organs/seedlings was found in roots. The circRNAs in seedlings, leaves and flowers originated mainly from genes involved in photosynthesis and the response to stimulus. The levels of circular and linear transcripts were not correlated. Although RNase R treatment enriches the analyzed RNA samples in circular transcripts, it may also have a negative impact on the stability of some of the circRNAs. We also showed that the normalization of NGS data by the library size is not proper for circRNAs quantification. Alternatively, we proposed four other normalization types whose accuracy was confirmed by ddPCR. Moreover, we provided a comprehensive characterization of circRNAs in *A. thaliana* organs and in seedlings. Our analyses revealed that plant circRNAs are formed in both stochastic and controlled processes. The latter are less frequent and likely engage circRNA-specific mechanisms. Only a few circRNAs were organ-specific. The lack of correlation between the accumulation of linear and circular transcripts indicated that their biogenesis depends on different mechanisms.

## Introduction

Circular RNAs (circRNAs) have recently emerged as a new, large class of noncoding RNAs with functions that, in most cases, remain unknown. To date, the abundance and evolutionary conservation of circRNAs have been shown across different species within the animal and plant kingdoms (Memczak et al., 2013; Salzman et al., 2013; Wang et al., 2014; Gao et al., 2015). However, more detailed studies of circRNAs thus far have only been performed in humans and several model animals [mainly cancer- and other disease-related studies (Qu et al., 2018)], leaving circRNAs in plants largely unexplored. CircRNAs have been identified in *Oryza sativa* (Lu et al., 2015; Ye et al., 2015), *Arabidopsis thaliana* (Liu et al., 2017), *Zea mays* (Chen et al., 2018), *Solanum lycopersicum* (Zuo et al., 2016), *Glycine max*, *Gossypium hirsutum* (Zhao et al., 2017), *Triticum aestivum* (Wang et al., 2016), *Hordeum vulgare* (Darbani et al., 2016), etc. A database listing circRNAs identified in plants has recently been created based on publicly available resources (Chu et al., 2017). Unfortunately, due to substantial differences in the procedures applied in the particular experiments (lack of standards in the analyses of circRNA), the generated data cannot be directly compared. For example, the number of circRNAs identified in different plants ranges from 62 in *H. vulgare* (Darbani et al., 2016) to approximately 10,000 in *S. lycopersicum* (Tan et al., 2017). The same phenomenon is also observed if only one plant species is considered; e.g., a number of circRNAs found in *A. thaliana* vary between 6 (Wang et al., 2014) and 6,012 (Ye et al., 2015), depending on the experimental approach. According to PlantcircBase (http://ibi.zju.edu.cn/plantcircbase/index.php; 1 March 2019), the total number of circRNAs in *A. thaliana* identified in different studies reached 38,938.

*A. thaliana* is currently the best-characterized plant in terms of circRNAs. A large fraction of these molecules was identified based on RNA-seq data generated earlier to study gene expression levels. Thus, RNA isolation and sequencing protocols were rarely optimized for circRNA research. During bioinformatics analyses, it was routinely assumed that a circRNA is valid if its presence is supported by at least two so-called back-spliced reads (reads overlapping a circRNA splicing site). The depth of the library was normally not taken into consideration, and thus, the numbers of circRNA candidates varied markedly between studies. Accordingly, questions about the levels of circRNA accumulation in plants and, consequently, their functional relevance have not been satisfactorily answered to date. Some reports have suggested that circRNAs in *A. thaliana* are expressed in tissue and developmental stage-specific manners (Sun et al., 2016; Chen et al., 2017; Liu et al., 2017). Additionally, Pan and coworkers (Pan et al., 2018) identified 1,583 heat-stress specific circRNAs that were absent in the control samples. In only a few cases, selected circRNAs identified based on RNA-seq data were experimentally validated by quantitative PCR (qPCR) (Wang et al., 2014; Liu et al., 2017; Cheng et al., 2018; Pan et al., 2018). Unfortunately, the obtained results were not used to optimize RNA-seq data normalization.

Critical review of the reports on the identification and differential accumulation of circRNAs in plants revealed that the published results are largely inconsistent due to the lack of standardized methods. To address this issue, we tested two major protocols of circRNA-seq library preparation (with and without RNase R treatment) and several approaches of RNA-seq data normalization that are routinely used to quantify other transcripts. We showed that the treatment of RNA samples with RNase R significantly increased the number of identified circRNAs. However, the spectrum of highly abundant circRNAs was reduced. Moreover, to compare the levels of circRNA accumulation in various plant organs, it is necessary to apply a circRNA-dedicated method of RNA-seq library normalization. After testing six normalization methods, we identified those that provided data that were most coherent with droplet digital PCR (ddPCR) results. We also noticed that the best correlation was observed when comparing samples collected from the same organs.

Next, using the optimized strategy, we investigated the accumulation of circular and corresponding linear transcripts in *A. thaliana* seedlings and organs, which led to the identification of ubiquitous and organ-specific circRNAs. Our results revealed that, in many cases, the abundance of circRNAs was not directly linked to the level of parent gene expression, which suggested the existence of circRNA-dedicated regulatory mechanisms.

## Materials and Methods

### Plant Material and RNA Extraction

Seeds of wild-type (Col-0) *A. thaliana* plants (deposition number: CS70000) were obtained from Arabidopsis Biological Resource Center (ABRC). The formal identification of the plant material used in the study was performed by J. Ecker laboratory (plant material donor) using Illumina sequencing by synthesis technology. Seeds were sterilized by placing them in filter tubes and washing them with 70% ethanol, distilled water, 0.01% Amistar 250 SC (Syngenta) and distilled water, using a vacuum pump. Next, the seeds were exposed to stratification for 4 days at 4°C in 0.1% agarose solution. Plants were grown using Arasystem (Betatech) and Jiffy-7 peat pellets in a growth chamber with 16 h of light at 23°C and 8 h of dark at 18°C. Whole seedlings at stage 1.0 (cotyledons fully open), whole roots and leaves at stage 3.90 (rosette growth complete) and whole flowers at stage 6.90 (flowering complete, flowers are no longer produced) (Boyes et al., 2001) were collected, frozen in liquid nitrogen and stored at −80°C until RNA isolation. Total RNA was isolated from 100 mg of powdered plant samples using the mirVana miRNA Isolation Kit (Thermo Fisher Scientific). For each replicate, 50 seedlings or 30 plants were used to obtain powdered samples. Subsequently, 10 μg of total RNA was treated with 2 U of Turbo DNase (Thermo Fisher Scientific) and purified with the QIAquick Nucleotide Removal Kit (Qiagen). RNA quality and integrity were determined using capillary electrophoresis (2100 Bioanalyzer, Agilent) with the Plant RNA Nano Assay. For further analyses, we used samples with the RNA integrity number (RIN) >9 for roots and flowers and >7.5 for tissues with a high content of chloroplast rRNA (seedlings and leaves).

### Library Preparation and Sequencing

Total RNA samples (isolated as described above) were divided into two portions: one portion was used to prepare an RNA-seq library without prior RNase R treatment (R−), and the other portion was used to prepare an RNA-seq library from RNase R-treated RNA (R+). For library preparation, we used 2.5 μg of total RNA. According to the first protocol (R−), 2.5 μg of total RNA was treated with Ribo-Zero (Illumina) following the manufacturer’s recommendation. The Ribo-Zero rRNA Removal Kit (Plant Leaf) was used for seedlings, leaves and flowers, and the Ribo-Zero rRNA Removal Kit (Plant Seed/Root) was used for roots. The level of rRNA depletion was determined by capillary electrophoresis (2100 Bioanalyzer, Agilent) with the Plant RNA Pico Assay.

The second protocol (R+) also included treatment of 2.5 μg of total RNA with Ribo-Zero. Then, 500 ng of the Ribo-Zero-treated RNA was denatured at 70°C for 3 min, treated with 10 U of RNase R (Epicentre) at 37°C for 1 h and subsequently purified with the QIAquick Nucleotide Removal Kit (Qiagen). The quality and integrity of the resultant RNA was tested using capillary electrophoresis (2100 Bioanalyzer, Agilent) with the Plant RNA Pico Assay.

One hundred nanogram aliquots of R− and R+ RNA samples from seedlings, roots, leaves, and flowers were prepared in four biological replicates. Next, 8 indexed libraries per biological replicate (16 in total) were prepared separately using the TruSeq RNA Sample Preparation Kit (Illumina) in accordance with the manufacturer’s instructions with minor changes. The protocol was modified by omitting the mRNA purification step and reducing reaction volumes by one-third at subsequent steps. The libraries were submitted to qualitative analysis using capillary electrophoresis (2100 Bioanalyzer, Agilent) with the High Sensitivity DNA Assay and quantitative analysis with a Qubit fluorometer (Invitrogen) prior to sequencing. The samples were sequenced with the Genome Analyzer IIx (Illumina) and 108-bp, paired-end protocol.

### CircRNA Identification

Raw reads were trimmed and quality-filtered with AdapterRemoval [version 1.5.4; (Schubert et al., 2016)]. As a result, remnant adapter sequences were removed, and read ends of low-quality were trimmed to save bases at the 3′ and 5′ end with a minimum quality score of 30 (99.9% base call accuracy). Reads shorter than 20 bases were removed. The rRNA content was assessed with SortMeRNA (version 2.1 [Kopylova et al., 2012)].

CircRNAs were identified using the protocol recommended by (Gao et al., 2015). Briefly, filtered reads were mapped to the reference genome (TAIR10) with BWA mem [version 0.7.10; (Li and Durbin, 2009)]. Subsequently, we used CIRI2 [version v2.0.5; (Gao et al., 2017)] and find_circ (Memczak et al., 2013) to identify the reads overlapping back-splicing sites. As a result of CIRI2, we obtained a list of circRNA candidates that were in summary supported by at least 2 back-spliced reads. CircRNAs not identified with find_circ were filtered out from final results.

For the selected circRNAs, their formation was confirmed by PCR. For each individual circRNA, two PCRs were performed: the first reaction involved divergent primers (circular transcript specific), and the second reaction involved convergent primers (linear transcript specific). As a control, analogous PCRs with gDNA as a template were performed. All primers are listed in **Additional file 1: Supplementary Table 1**. Prior to PCR, cDNA was obtained in the reverse transcription (RT) reaction, including 1 μg of Turbo DNase-treated total RNA and iScript Reverse Transcription Supermix (Bio-Rad). PCR mixtures (50 μl of the total volume) contained 1 μl of cDNA template from the 20-μl RT reaction or 10 ng of gDNA, 1.25 U of Taq DNA polymerase (5 U/µl), 1,75 mM MgCl_2_, 0.2 mM dNTPs, 1× Taq Buffer with (NH_4_)_2_SO_4_ (Thermo Fisher Scientific), and 0.3 µM of each primer (Genomed). PCR was performed in a T-100 thermal cycler (Bio-Rad) using the following program: preheating at 95°C for 3 min, followed by 40 cycles (with divergent primers) or 35 cycles (with convergent primers) at 95°C for 30 s, 59°C to 62°C (dependent on the melting temperature (T_m_) of the primers used – listed in **Additional file 1: Supplementary Table 1**) for 30 s and 72°C for 30 s. Finally, the reaction mixtures were subjected to elongation at 72°C for 5 min. PCR products were purified using the QIAquick PCR Purification Kit (Qiagen), resolved in 2% agarose gel for 70 min at 120 V in a Wide Mini-Sub Cell GT System (Bio-Rad) and poststained with 0.5 µg/ml EtBr solution for 20 min on a rocker. Gel images were obtained with a ChemiDoc XRS+ System (Bio-Rad) and analyzed with Image Lab Software (Bio-Rad). Purified PCR products were cloned using the TOPO TA Cloning Kit (Thermo Fisher Scientific) in accordance with the manufacturer’s instructions. Clones were screened by EcoRI digestion (Thermo Fisher Scientific), and positive clones were purified using the QIAquick PCR Purification Kit (Qiagen) and sequenced using the 3130XL Genetic Analyzer (Applied Biosystem). The sequencing results were analyzed using SnapGene software (GSL Biotech LLC).

### Quantitative Analysis of circRNA

The quantity of each circRNA was estimated based on the normalized back-spliced read count. Six normalization methods were tested: by the library size, by the sum of back-spliced reads, by the DESeq2 size factor, by the library size without rRNA and chloroplast RNA reads, by the sum of back-spliced reads without rRNA and chloroplast RNA reads and by the number of raw reads mapping to the endogenous marker *ACT2*. To determine the raw read numbers mapping to the selected gene, we applied the protocol proposed by (Pertea et al., 2016): filtered reads were mapped with HISAT2 (version 2.1.0; [Kim et al., 2015)] to the reference *A. thaliana* genome (TAIR10). Gene quantification analyses were performed with StringTie [version 1.3.3b; (Pertea et al., 2015)].

The distribution of circRNAs in organs and seedlings and the circRNA content in biological replicates were visualized using the VennDiagram R package. Heatmaps and pairwise comparison scatterplots were produced using the ggplot2 R package. The circRNA accumulation pattern in organs/seedlings on heatmaps was visualized with 4 discrete classes (high, medium, low, not detected). CircRNAs were grouped by hierarchical agglomerative clustering and divided between classes as follows: high for 5% of circRNAs with the highest accumulation in organs/seedlings; medium - circRNAs with higher accumulation than 80% of circRNAs and lower than 5% of circRNAs with the highest accumulation; low – remaining circRNAs; not detected - circRNAs not detected in particular organ/seedlings. Linear counterparts were ordered corresponding to circRNAs. To evaluate associations between two variables (circRNAs and their linear counterparts; accumulation of circRNA obtained from RNA-seq and ddPCR) we calculated Pearson correlation using R package. GO enrichment analyses were conducted with agriGO (Tian et al., 2017).

ddPCR reactions were performed with the QX200 ddPCR System (Bio-Rad) in accordance with the manufacturer’s protocol. ddPCR reactions were performed with two sets of primers: a pair of divergent primers or a pair of convergent primers. All primers used are listed in **Additional file 1: Supplementary Table 1**. Assay mixtures contained 1 μl of cDNA template (obtained as described above), 10 μl of QX200 ddPCR EvaGreen Supermix (Bio-Rad), and 0.1 µM of each primer (Genomed). Thermal cycling conditions in the T-100 thermal cycler (Bio-Rad) were as follows: 95°C for 5 min, followed by 40 cycles of 95°C for 30 s and 59°C to 62°C (dependent on the T_m_ of the primers used – listed in **Additional file 1: Supplementary Table 1**) for 1 min, a final inactivation step at 4°C for 5 min followed by 90°C for 5 min and an infinite hold at 12°C. All steps were performed at a ramp rate of 2°C/s. The read-out was performed with the QX200 Droplet Reader (Bio-Rad). The results were analyzed using the QuantaSoft Analysis Pro software (Bio-Rad). Each experiment was carried out in three independent biological replicates. To exclude variances in the RT reaction between different templates, for each biological replicate, the *ACT2* expression level was determined in parallel in four technical repeats as a reference for normalization.

## Results

### CircRNA Identification in *A. thaliana* Seedlings and Organs

Considering the problems encountered by other researchers studying circRNA in plants, we attempted to evaluate and optimize the circRNA-dedicated protocols for RNA-seq and sequencing data analysis. Our efforts were focused on RNA-seq because it is currently the only method that can provide a comprehensive landscape of circRNAs in the whole plant and its particular organs. In the first step, we isolated total RNA from seedlings, roots, leaves, and flowers of wild-type *A. thaliana* (Columbia ecotype, abbreviated Col-0) and prepared RNA-seq libraries using two protocols that are widely applied in circRNA studies. In line with the first approach, we used total RNA depleted of rRNA (samples contained both linear and circular transcripts and resultant libraries were denoted with R−, see **Additional file 1: Supplementary Table 2A**; samples from leaves and seedlings contained also traces of chloroplast rRNA, see **Additional file 2: Supplementary Figure 1**). In accordance with the second approach, we used total RNA depleted of rRNA and subsequently treated with RNase R (samples contained circRNA and traces of linear RNA, and resultant libraries were designated with R+, see **Additional file 1: Supplementary Table 2B**). All RNA-seq experiments were performed in 4 biological replicates.

On average 60,292,166 paired-end reads were obtained for R− libraries; 99.54% of the reads passed trimming and quality filtration, and 95.30% of them mapped to the *A. thaliana* reference genome (TAIR10). For the R+ libraries, 25,932,926 paired-end reads, on average, were obtained; 99.00% of the reads passed trimming and quality filtration, and 91.87% of them mapped to the *A. thaliana* reference genome (TAIR10).

For the R− libraries, from 0.6% to 7.57% of the reads mapped to the rRNA sequences (roots and leaves, respectively). For the R+ libraries, the percentage of reads that mapped to the rRNA sequences varied from 0.27% in roots to 3.18% in flowers. This observation was consistent with the capillary electrophoresis results (**Additional file 2: Supplementary Figure 1**).

For circRNA discovery, we initially used CIRI2 (Gao et al., 2015) as the best-performing *de novo* identifying program (Szabo and Salzman, 2016; Hansen, 2018). In total, we detected 5,235 and 15,253 distinct circRNA candidates in R− and R+, respectively, supported by at least two back-spliced reads (**Additional file 1: Supplementary Tables 3A, B**). A large fraction of the detected circRNA candidates was supported by 2 to 5 back-spliced reads (4,297 circRNAs = 82.1% in R− and 12,155 circRNAs = 79.6% in R+). However, we also identified 39 (R−) and 88 (R+) circRNAs supported by more than 100 back-spliced reads. Approximately half of the identified circRNAs had been reported previously, 37.7% and 24.8% [AtCircDB (Ye et al., 2017)], and 42.5% and 28.9% [PlantcircBase, (Chu et al., 2017)] in R− and R+, respectively. In general, the majority of circRNAs (R−: 88.2%, R+: 97.3%) were derived from annotated genes. Considering that we only found 4,619 (R−) and 14,848 (R+) genes producing circRNAs, the median number of circRNAs per gene was 1. However, for 2 genes, more than 10 circRNAs were found in both libraries. A circRNA typically overlapped 1 to 4 exons (1 exon 31.5% and 28.3%; 2 exons 32.8% and 32.9%; 3 exons 15.9% and 19.8%; and 4 exons 8.6% and 9.4% in R− and R+, respectively), but we also identified 24 (R−) and 36 (R+) circRNA candidates enclosing more than 10 exons.

**Figure 1:**
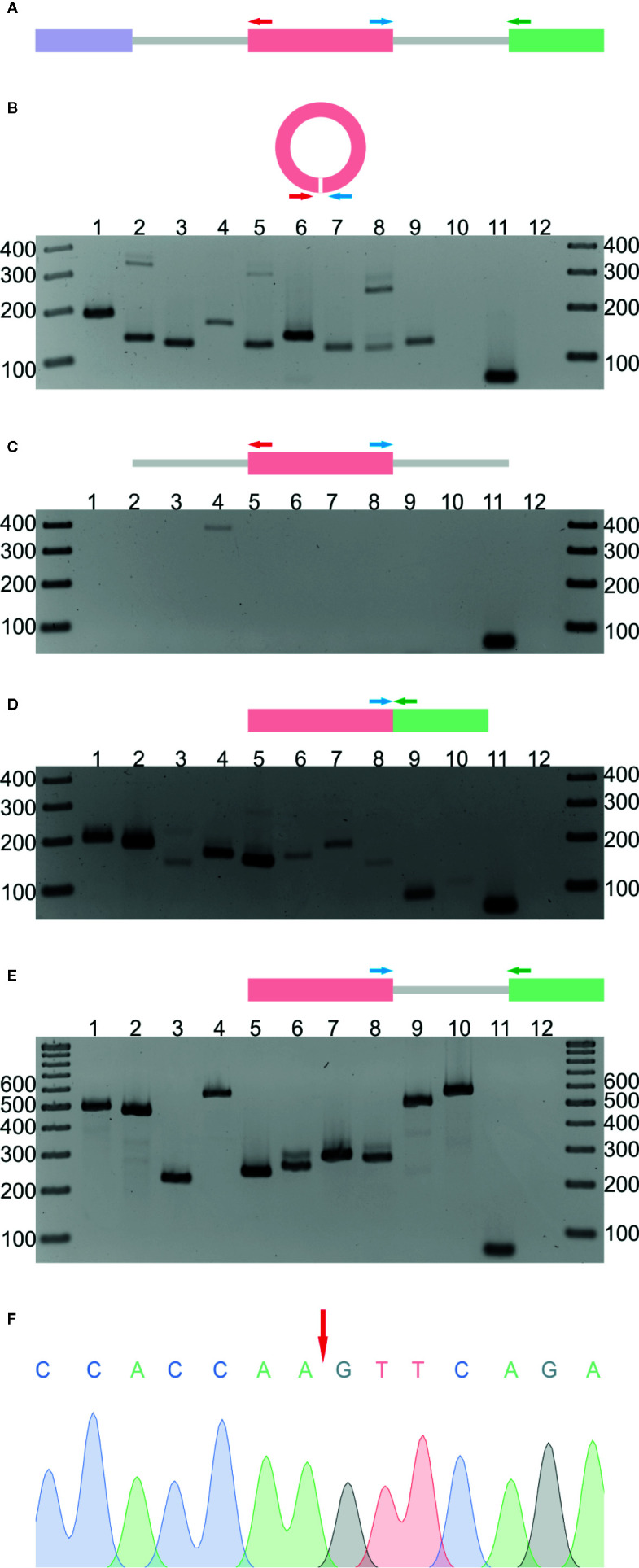
Validation of 10 selected circRNAs identified in *A. thaliana*. **(A)** Schematic representation of the PCR strategy. Colored rectangles and gray lines represent exons and introns, respectively. Divergent (red and blue arrows) and convergent (blue and green arrows) primers were used to detect circular RNAs and their linear counterparts. **(B)** Detection of circRNAs by RT-PCR with leaf cDNA as template and divergent primers. Lanes 1 to 10 show PCR products corresponding to circRNA candidates. Lanes 11 and 12 represent positive (leaf cDNA template) and negative (no template) controls, respectively, with convergent primers for *ACT2*. **(C)** PCR products obtained with leaf gDNA and divergent primers. Lanes 1 to 10 show PCR products corresponding to circRNA candidates. Lanes 11 and 12 represent positive (leaf cDNA template) and negative (no template) controls, respectively, with convergent primers for *ACT2*. **(D)** Detection of linear transcripts by RT-PCR with leaf cDNA as template and convergent primers. Lanes 1 to 10 show PCR products corresponding to linear counterparts of circRNA candidates. Lanes 11 and 12 represent positive (leaf cDNA template) and negative (no template) controls, respectively, with convergent primers for *ACT2*. **(E)** PCRs with leaf gDNA and convergent primers. Lanes 1 to 10 show PCR products corresponding to linear counterparts of circRNA candidates. Lanes 11 and 12 represent positive (leaf cDNA template) and negative (no template) controls, respectively, with convergent primers for *ACT2*. **(F)** Sequencing chromatogram of the circRNA 1 (5:1106879-1107381) amplification product. The red arrow indicates the back-splice site. The uncropped gel images are shown in **Additional file 2: Supplementary Figure 2**. Additional Sanger sequencing results are shown in **Additional file 2: Supplementary Figure 3**. CircRNA IDs: 1) 5:1106879-1107381; 2) 1:382803-383130; 3) 2:19672192-19672380; 4) 1:30349032-30349237; 5) 2:14529841-14530023; 6) 4:13026572-13027129; 7) 3:3172073-3172253; 8) 2:16614068-16615133; 9) 2:17272368-17272938; 10) 1:3674466-3675958.

In the next step, the RNA-seq results were validated by reverse transcription PCR (RT-PCR). We selected 10 circRNA candidates that were identified in both the R− and R+ libraries. Nine of the candidates were found in leaves, and 1 was absent in leaves but present in 2 other organs and in seedlings (**Additional file 1: Supplementary Tables 3A, B**). As a template in the RT-PCR reaction, we used total RNA from leaves (neither depleted of rRNA nor treated with RNase R). Regions of circRNAs encompassing back-splice junctions were amplified using circRNA-specific divergent primers (each pair specific for an individual circRNA, for details, see **Additional file 1: Supplementary Table 1**, **Figure 1A**). In nine cases, RT-PCR products of the predicted lengths were obtained (**Figure 1B**). The product was not observed, as expected, for the circRNA that, according to the RNA-seq data, was absent in leaves (lane 10). Three randomly selected products were cloned and sequenced, which confirmed the presence of back-splice junctions (**Figure 1F**, **Additional file 2: Supplementary Figures 3A, B**, **Supplementary Figure 4B**). To rule out the possibility of genomic rearrangements, we performed PCR involving the same set of divergent primers and genomic DNA (gDNA) as a template (**Figure 1C**). No product was obtained except in one reaction (lane 4). Cloning and sequencing of this product showed that it resulted from mispriming to the AT5G37790 locus (**Supplementary Figure 3C**). In addition, to confirm the formation of linear counterparts of all circRNAs and to show that the gDNA contained the expected arrangement of exons, we performed PCR using, respectively, complementary DNA (cDNA) (**Figure 1D**) and gDNA (**Figure 1E**) as templates and convergent primers (each pair specific for the individual transcript/gene) flanking the canonical splice junctions located downstream of the circRNA-producing exon (**Additional file 1: Supplementary Table 1**, **Figure 1A**). As controls, we performed PCR with convergent primers specific for *ACTIN2* (*ACT2*, AT3G18780). In the positive control (lane 11), leaves cDNA were used as a template, and in the negative (lane 12), no template was added. The obtained results were fully consistent with the RNA-seq data obtained for both the R− and R+ libraries.

**Figure 2:**
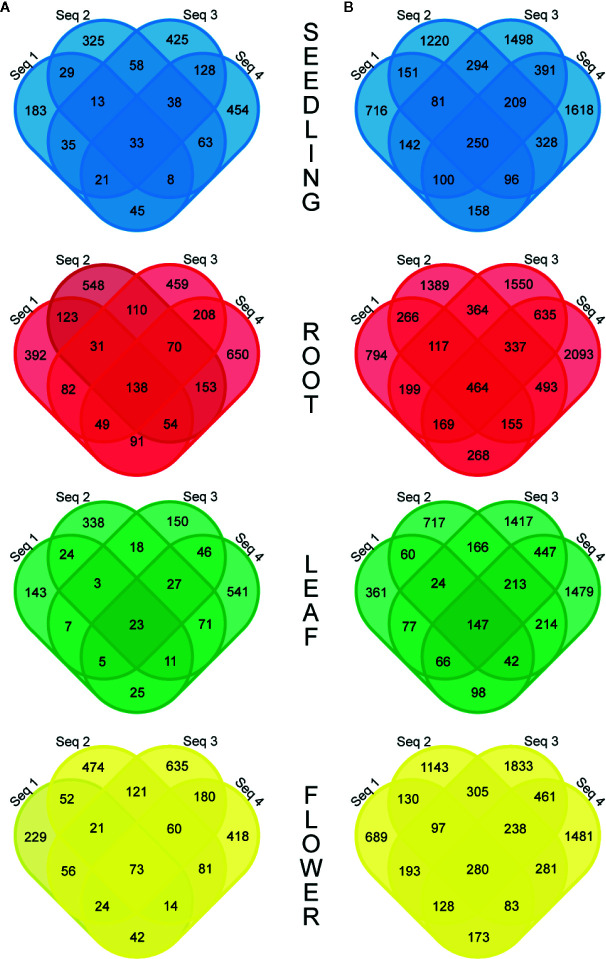
Venn diagrams showing the number of common circRNAs detected in four biological replicates in the R− **(A)** and R+ **(B)** libraries.

More detailed investigations of the RNA-seq dataset revealed that the fraction of circRNAs identified in four biological replicates was surprisingly small and varied from 1.3% (R−) to 2.7% (R+) for the leaf libraries and from 4.4% (R−) to 5.0% (R+) for the root samples (**Figure 2**). Interestingly, some circRNAs supported by back-spliced reads in the R− libraries were not present in the R+ libraries (**Additional file 1: Supplementary Table 3C**). To confirm their presence, we selected 7 circRNAs identified by RNA-seq analysis only in R− library. Five of the circRNAs were identified in R− and not identified in R+ libraries from leaves and 2 were absent in leaves but identified in R− libraries from seedlings and roots (**Additional file 1: Supplementary Table 3C**). In addition, we selected a circRNA candidate derived from the chloroplast chromosome that was found in both R− and R+ libraries from leaves (**Additional file 1: Supplementary Tables 3A, B**). Then, we used standard RT-PCR to determine whether they were present in total RNA extracted from leaves. In the PCR, we used cDNA (obtained as described in Materials and Methods) and divergent primers specific for individual circRNAs (**Additional file 1: Supplementary Table 1**). As in the former experiment, primers specific for *ACT2* were used in control reactions: positive included cDNA from leaves and negative contained no template. In 7 reactions, the products of predicted lengths were obtained (**Additional file 2: Supplementary Figure 4A**, lanes 1–7). Surprisingly, the circRNA identified only in roots was amplified (**Additional file 2: Supplementary Figure 4A**, lane 4). Nevertheless, the product for circRNA identified only in seedlings was not formed (**Additional file 2: Supplementary Figure 4A**, lane 8). PCR product from lane 3 was cloned and sequenced, which confirmed the presence of back-splice junction (**Additional file 2: Supplementary Figure 4B**). These results provided an additional piece of evidence that circRNAs not found in R+ libraries were present *in planta*. Additionally, in this experiment, we confirmed the formation of the circRNA candidate derived from the chloroplast chromosome that was found in both R− and R+ libraries from leaves (**Additional file 2: Supplementary Figure 4A**, lane 7).

The above results showed that a large fraction of circRNAs did not form reproducibly in plants. Moreover, some circRNAs with considerable functional potential, i.e., accumulating in plants to high levels, were not identified in R+ libraries. Consequently, in further analyses, we used RNA-seq data obtained with R− libraries, as we intended to study highly abundant, reproducible circRNAs with functional potential.

### Quantitative Analysis of circRNA in Seedlings and Plant Organs

In former reports on circRNAs, their accumulations in whole plants and in different organs were usually determined based on RNA-seq data (read counts) without further validation by other experimental methods. To obtain detailed insight into this issue, we compared the results of RNA-seq-based quantitative analysis of circRNAs with corresponding data obtained using ddPCR. To achieve this goal, we selected 8 circRNAs and determined their levels in seedlings, roots, leaves, and flowers by ddPCR. The summarized ddPCR results based on 3 biological replicates are shown in **Table 1** col. 1 (for details see **Additional file 1: Supplementary Table 4**). These results were used as a reference for determining the best method of RNA-seq data normalization. First, the back-spliced read counts obtained for the individual circRNA were normalized using the total number of reads in a library (**Table 1**, col. 2), as this type of normalization is the most common in circRNA studies (Ye et al., 2015; Wang et al., 2016; Liu et al., 2017). The result of Pearson correlation coefficient calculated for this dataset was 0.67. Thus, the normalization by the library size did not provide satisfying results. Next, the read counts were normalized using the total number of back-spliced reads in the library (**Table 1**, col. 3). This approach provided better results, with the Pearson correlation coefficient reaching 0.77. Finally, the read counts were normalized using the size factors calculated by the DESeq2 program (**Table 1**, col. 4). This type of normalization is frequently used to analyze differential gene expression (see Materials and Methods). The obtained correlation coefficient was 0.76.

**Table 1:** Abundances of circRNAs in seedlings and plant organs determined by ddPCR and RNA-seq with different types of data normalization (for the extended version of this table, see **Additional file 1: Supplementary Table 4**).

**Column number**			**1**		**2**	**3**	**4**	**5**	**6**	**7**
**Sample**			**ddPCR**		**RNA-seq normalized reads**					
circRNA name	circRNA ID	Organ	cp/µg	st.dev.	Library size	Back-spliced reads	DESeq2 size factor	Library size—without rRNA and chloroplast reads	Back-spliced reads—without rRNA and chloroplast reads	ACT2 (AT3G18780)
A	4:4196362-4197112	Seedling	70.9	42.5	15.5	299028.5	1256309309.2	34.1	416842.1	32441.5
B	5:1106879-1107381	Seedling	75.9	46.3	32.5	639122.0	2401751918.5	56.9	846227.8	62709.9
C	1:382803-383130	Seedling	6.3	5.6	4.6	99458.9	392892148.2	8.1	139727.3	10254.1
D	2:19672192-19672380	Seedling	8.1	2.3	3.8	75095.2	350726157.6	7.8	103507.7	9207.0
E	1:30349032-30349237	Seedling	6.6	7.3	4.2	85267.6	325177084.7	8.2	117946.2	8406.7
F	2:14529841-14530023	Seedling	4.4	3.9	1.8	32451.1	107648015.3	3.5	41692.9	2756.1
G	3:3172073-3172253	Seedling	8.6	7.7	1.0	17127.8	86565020.0	2.7	23909.8	2232.6
H	2:16614068-16615133	Seedling	57.5	36.6	2.2	42413.1	152047044.7	3.9	56545.5	3941.6
A	4:4196362-4197112	Root	310.8	58.0	62.8	514242.3	1459154931.5	64.3	515369.2	40115.6
B	5:1106879-1107381	Root	403.9	35.5	116.7	984781.5	2670437284.5	119.4	986902.9	73977.9
C	1:382803-383130	Root	50.8	8.1	19.8	169589.9	442625591.2	20.2	169930.3	12169.8
D	2:19672192-19672380	Root	42.0	8.0	15.8	136481.1	358654044.8	16.2	136774.5	10013.7
E	1:30349032-30349237	Root	8.5	3.7	15.1	136987.2	328463657.3	15.3	137248.5	9118.7
F	2:14529841-14530023	Root	1.7	3.0	0.4	2450.5	7547596.9	0.4	2458.2	223.8
G	3:3172073-3172253	Root	6.7	0.6	4.1	35013.7	84508071.1	4.2	35082.9	2362.5
H	2:16614068-16615133	Root	165.7	45.2	1.4	11901.1	30190387.4	1.5	11931.7	895.0
A	4:4196362-4197112	Leaf	712.3	103.7	30.1	1260098.8	4104055169.7	58.6	1358140.6	127464.0
B	5:1106879-1107381	Leaf	751.1	177.7	33.8	1561949.7	4696612483.6	70.4	1650893.6	145891.2
C	1:382803-383130	Leaf	87.5	23.4	5.5	189330.0	568864974.2	8.8	217091.3	17681.0
D	2:19672192-19672380	Leaf	43.8	9.6	2.9	121121.1	355550976.4	5.4	133771.1	11041.2
E	1:30349032-30349237	Leaf	14.2	12.4	3.2	107435.2	392817341.8	5.3	122590.9	12181.3
F	2:14529841-14530023	Leaf	21.5	18.4	1.9	86108.0	186248886.7	3.5	94131.7	5776.3
G	3:3172073-3172253	Leaf	13.2	5.6	0.8	35715.0	60958705.1	1.3	40667.9	1886.3
H	2:16614068-16615133	Leaf	413.2	111.2	3.1	151191.6	399562884.5	6.4	159512.9	12416.1
A	4:4196362-4197112	Flower	966.3	186.2	50.6	518142.0	1815139368.8	65.4	547677.8	52437.1
B	5:1106879-1107381	Flower	1095.2	233.1	104.2	1150954.9	3452046537.0	129.6	1215016.1	99180.1
C	1:382803-383130	Flower	128.6	48.6	11.7	127251.5	403191198.6	14.8	135097.2	11576.1
D	2:19672192-19672380	Flower	42.0	17.0	6.7	79017.3	206880247.3	8.0	83020.7	5931.3
E	1:30349032-30349237	Flower	16.0	9.0	11.6	141156.9	382052606.6	13.5	149205.6	11003.1
F	2:14529841-14530023	Flower	5.6	9.7	5.1	61782.3	155535658.2	6.1	65279.9	4431.7
G	3:3172073-3172253	Flower	12.2	7.2	4.0	42481.2	144215416.7	5.1	44790.1	4178.8
H	2:16614068-16615133	Flower	581.3	96.7	9.3	107236.4	329206126.5	11.1	112814.1	9570.6
Correlation ddPCR vs RNA-seq				All samples	0.67	0.77	0.76	0.76	0.75	0.77
				Seedling	0.75	0.75	0.76	0.78	0.75	0.76
				Root	0.90	0.89	0.90	0.90	0.89	0.90
				Leaf	0.92	0.92	0.92	0.92	0.92	0.92
				Flower	0.87	0.85	0.88	0.87	0.85	0.88

The difficulties with proper RNA-seq data normalization motivated us to compare the content of the RNA-seq libraries. We found significant differences in the composition of organ/seedling transcriptomes. In particular, the numbers of reads mapping to rRNA genes and the chloroplast genome were markedly distinct (**Additional file 1: Supplementary Table 2**). This phenomenon is the most likely explanation for the ineffective normalization based on the library size. Consequently, we excluded from the libraries the reads from rRNA and chloroplast RNA. We normalized this new set of RNA-seq data and again compared them with the results of the ddPCR-based analysis (**Table 1**, col. 5 and 6). The correlation coefficient reached 0.76. Thus, the transcriptome composition is an important factor affecting the results of quantitative RNA-seq-based analysis of circRNA. However, rRNA and chloroplast-derived reads were not solely responsible for the differences.

Similar results were obtained when we used reference genes as an internal, steady factor to normalize the RNA-seq data. The best results were obtained for *ACT2* gene (**Table 1**, col. 7), which is known to be expressed at a relatively stable level throughout the whole plant (Dheda et al., 2004; Radonic et al., 2004). In this case, the Pearson correlation coefficient was 0.77, (**Table 1**, col. 3). It is noteworthy that the Pearson correlation coefficient between the ddPCR and normalized RNA-seq results was high if a single organ was considered regardless of the normalization type applied. The correlation values ranged from 0.88 (flowers) to 0.90 (roots) and 0.92 (leaves). The correlation coefficient was relatively lower (0.76) only for seedlings, which might be explained by the generally low abundances of circRNAs in the developing plant (**Table 1**).

The results obtained indicated that four of tested normalization methods, namely, normalization by: (i) the number of back-spliced reads, (ii) the DESeq2 size factor (iii) the library size without rRNA and chloroplast RNA reads, (iii) the number of back-spliced reads without rRNA and chloroplast RNA reads and (iv) housekeeping gene *ACT2*, reached a similar Pearson correlation coefficient between the ddPCR and RNA-seq results of 0.76 to 0.77 and might be used alternatively.

Moreover, analysis of the ddPCR results showed that 50 copies of circRNA per microgram of total RNA (cp/µg) represented the minimal value for quantification of circRNA using this method. Below this threshold, the standard deviation and average values were comparable, which made the results unreliable (**Additional file 1: Supplementary Table 4**).

**Figure 3:**
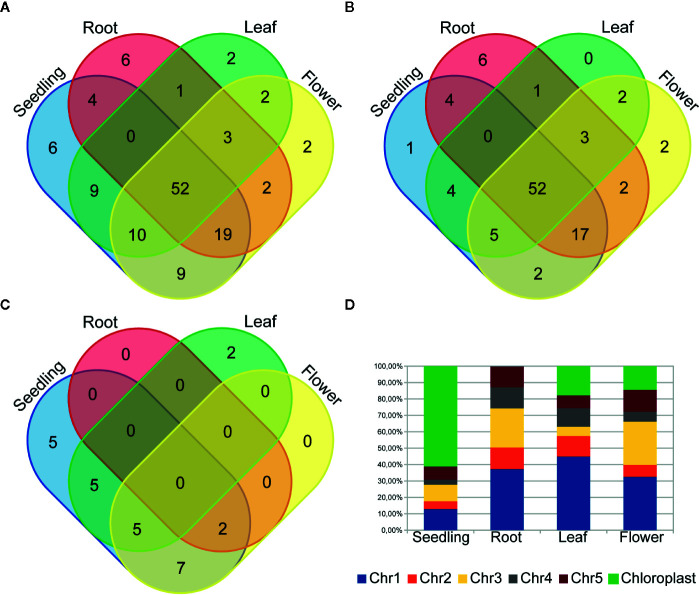
Venn diagrams showing the circRNA number in plant seedling/organs from **(A)** chromosomes 1-5 and the chloroplast genome, **(B)** only chromosomes 1-5 and **(C)** only the chloroplast genome. **(D)** The distribution of back-spliced reads supporting circRNAs among chromosomes.

To examine circRNA differential accumulation using RNA-seq data, we applied normalization by the library size without reads that mapped to the chloroplast genome and rRNA genes. Our analysis included only those circRNAs that were represented by at least 10 RNA-seq normalized reads (average of four biological replicates), which corresponded to the threshold of 50 cp/µg established by the ddPCR and were additionally identified by find_circ program (**Additional file 1: Supplementary Table 5**). One hundred twenty seven circRNA candidates met this criterion; 109, 87, 79 and 99 circRNAs in seedlings, roots, leaves, and flowers, respectively. One hundred twelve (88.2%) and 67 (52.8%) of them were previously identified and deposited in online databases (PlantcircBase and AtCircDB respectively). Fifty two (40.9%) circRNAs were present in all 3 organs and in seedlings. The largest number of unique circRNAs (8 with and 6 without chloroplast circRNAs) was found in roots (**Figures 3A, B**). CircRNAs derived from the chloroplast genome were present in seedlings (24), roots (2), leaves (12) and flowers (14). Only 7 of the circRNAs were common to all organs and seedlings. Five chloroplast circRNAs were seedling-specific, 2 leaf-specific, whereas there were no root- and flower-specific ones (**Figure 3C**).

### *A. thaliana* Genes Giving Rise to circRNAs

Gene ontology (GO) analysis of chloroplast genes giving rise to circRNAs did not reveal any significant enrichment. Consecutive GO analysis revealed that the circRNAs in seedlings, leaves and flowers predominantly came from genes involved in photosynthesis and the response to stimulus (seedling and flower) (p-value: <0.01), **Additional file 1: Supplementary Tables 6A–C** respectively. Statistically significant GO enrichment was not observed for genes producing circRNAs in roots.

The analysis of back-spliced reads revealed the predominance of reads corresponding to chloroplast circRNAs in seedlings (**Figure 3D**). We also attempted to determine if any *A. thaliana* chromosomes were a preferable source of circRNAs. However, this analysis did not reveal any statistically significant differences.

**Figure 4:**
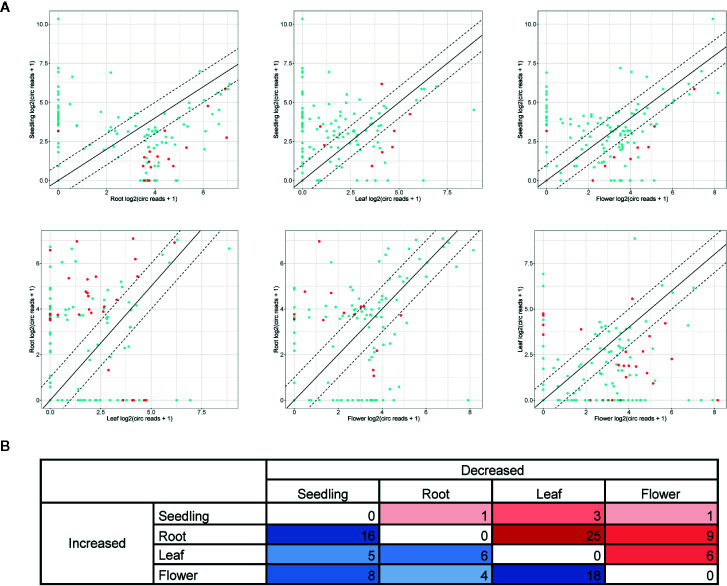
**(A)** Pairwise comparison of circRNA accumulation in seedlings, roots, leaves, and flowers. Dashed line: two-fold cutoff; comparisons with p-value ≤ 0.05 marked in red. **(B)** The numbers of circRNAs with at least a two-fold increase or decrease in accumulation level between sample types (p-value ≤ 0.05).

Further analyses were focused on circRNAs that were present in at least two organs or in one organ and seedlings. A pairwise comparison revealed an organ-specific pattern of circRNA accumulation (**Figure 4A**, **Additional file 1: Supplementary Table 5**). The largest number of circRNAs with significantly increased accumulation (p-value ≤ 0.05) in comparison to other organs/seedlings was found in roots (16, 25 and 9 circRNA vs seedlings, leaves, and flowers, respectively, **Figures 4A, B**). Cellular process and organelle part GO terms (p-value <0.000001) were the only categories characteristic for the group of genes encoding differentially expressed circRNAs in leaves versus flowers (24, **Additional file 1: Supplementary Table 6D**). No other statistically significant GO enrichment was observed. The smallest number of circRNAs with increased accumulation was identified in seedlings (1, 3 and 1 for seedlings vs roots, leaves, and flowers respectively, **Figure 4B**).

### Accumulation of circRNAs and Their Linear Counterparts

**Figure 5:**
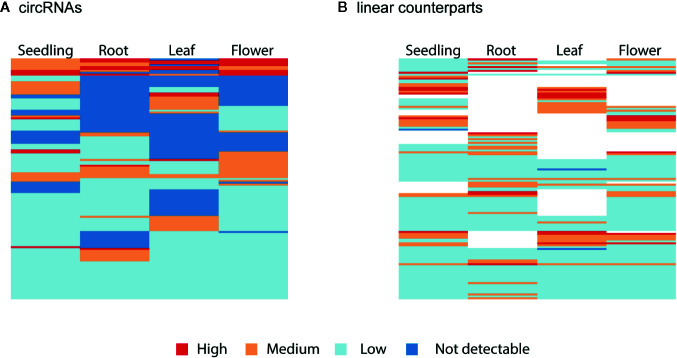
Clustered heatmaps showing the accumulation pattern of the **(A)** most abundant circRNAs and **(B)** their linear counterparts in matching order. The Pearson correlation coefficient between circRNAs and linear transcripts was 0.46. When circRNA was not detected (dark blue), the linear isoform abundance was not assessed (white).

**Figure 6:**
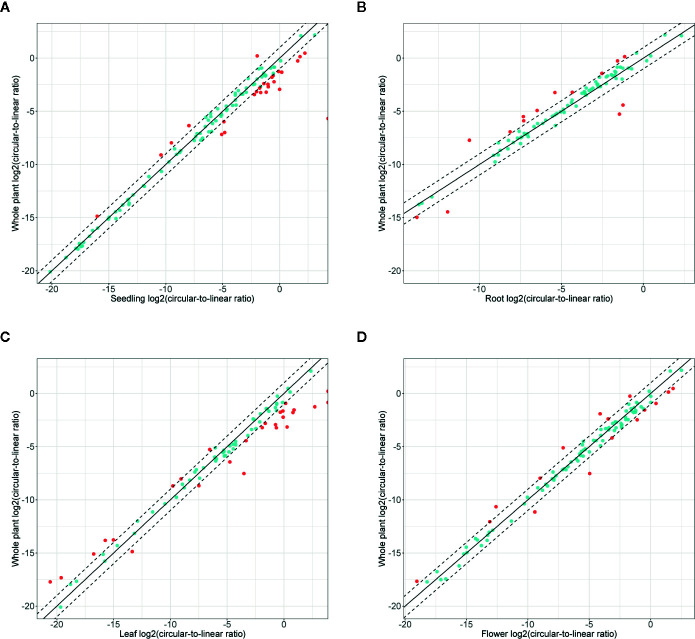
Accumulation of circRNA in the whole plant (seedling and all organs) versus **(A)** seedlings, **(B)** roots, **(C)** leaves, and **(D)** flowers. Dashed line: two-fold cutoff.

Finally, we studied the correlation between the abundance of circRNAs and their linear counterparts. The expression profiles of circular and linear transcripts in different organs/seedlings arranged in a matching order are shown in **Figures 5A, B**, respectively. The Pearson correlation coefficient between the accumulation of corresponding circular and linear RNAs was 0.46 (0.52, 0.04, 0.09, and 0.36 in seedlings, roots, leaves, and flowers, respectively). Thus, in most cases, there was no relationship between the levels of circRNA and linear counterpart production. For 6 circRNAs, their accumulation levels were at least 1.5 times higher than those observed for the corresponding linear transcripts (in at least one organ/seedling; **Additional file 1: Supplementary Table 5**). Four originated from intergenic regions, and 2 (1:12195268-12196524, 3:17169794-17170903) were from transcripts of genes AT1G33615 and AT3G46614 (encodes ncRNA). Abundance of circRNAs was strongly enriched in leaves and seedlings compared with their linear counterparts (**Figure 6**). In roots, and flowers, the ratios between the levels of circRNAs and corresponding linear RNAs were similar. The results of organs/seedlings pairwise comparisons of circular to linear ratios are presented in **Additional file 2: Supplementary Figure 5**.

**Figure 7:**
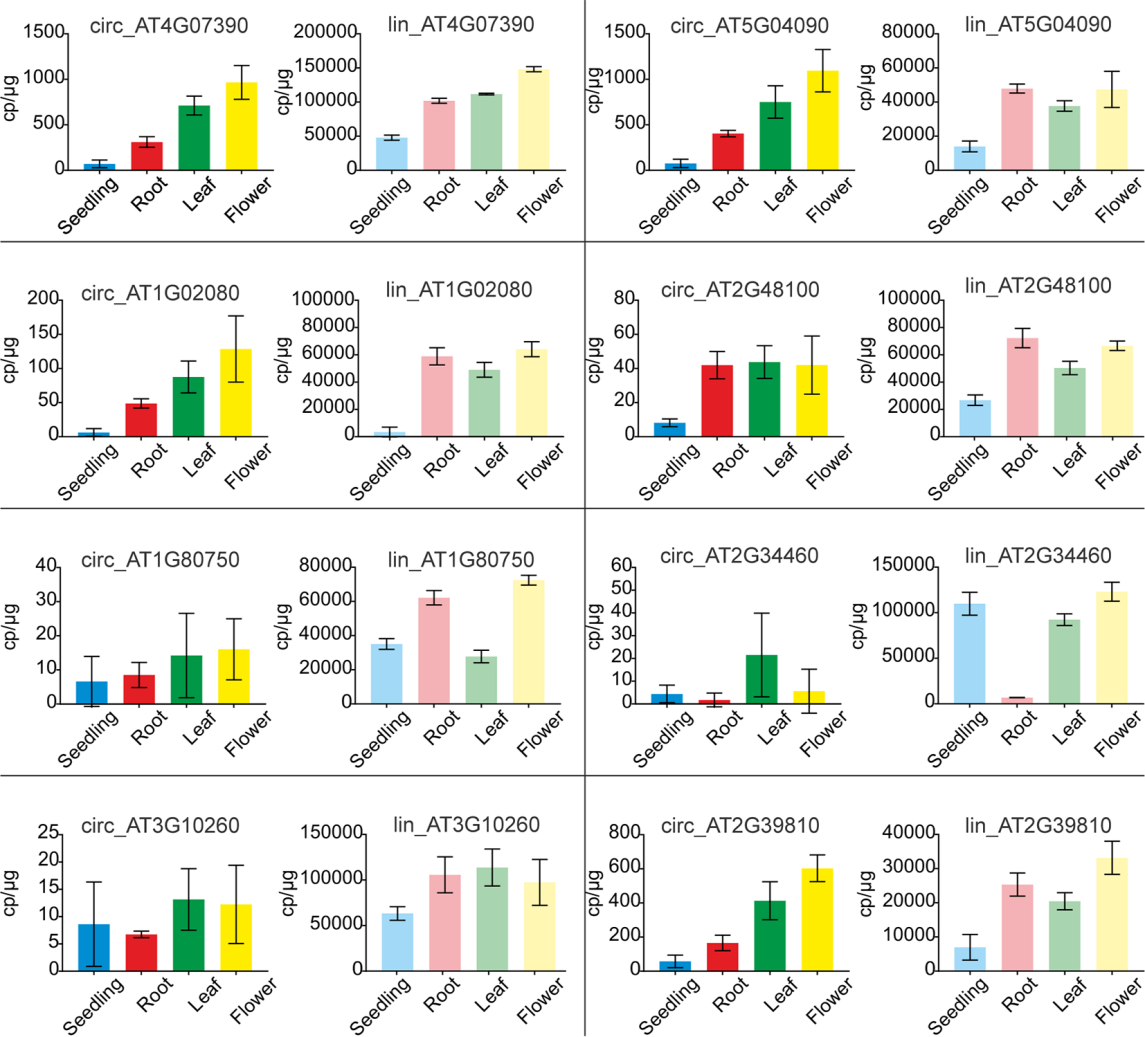
Quantification of circRNA and their linear counterpart transcripts by ddPCR. Graphs with ddPCR results for circular RNAs (bright colors) and their linear counterparts (faded colors) are presented adjacent to one another. For circular and linear transcript quantification, sets of divergent and convergent primers were used, respectively. Each experiment was carried out in three independent biological replicates. CircRNA IDs: circ_AT4G07390: 4:4196362-4197112; circ_AT5G04090: 5:1106879-1107381; circ_AT1G02080: 1:382803-383130; circ_AT2G48100: 2:19672192-19672380; circ_AT1G80750: 1:30349032-30349237; circ_AT2G34460: 2:14529841-14530023; circ_AT3G10260: 3:3172073-3172253; circ_AT2G39810: 2:16614068-16615133.

To verify the above observations, the levels of circRNAs and corresponding linear transcripts accumulation in seedlings, roots, leaves, and flowers were precisely determined with ddPCR. To this end we selected 8 pairs of circular-linear RNAs. The most abundant circRNAs were circ_AT4G07390 and circ_AT5G04090, followed by circ_AT2G39810, circ_AT1G02080, circ_AT2G48100, circ_AT2G34460, circ_AT1G80750, and circ_AT3G10260. Four of the tested circRNAs (circ_AT4G07390, circ_AT5G04090, circ_AT1G02080, and circ_AT2G39810) displayed a common accumulation profile, characterized by a continual increase in their amounts from seedlings through roots, leaves to flowers (**Figure 7**). In the case of circ_AT4G07390 and circ_AT2G39810, there was at least a 2-fold increase in the accumulation levels between seedlings and roots, as well as between roots and leaves, with a further increase in flowers. The other 4 analyzed circRNAs (circ_AT2G48100, circ_AT1G80750, circ_AT2G34460, and circ_AT3G10260) were rare, and in general, they did not reach 50 cp/µg. Therefore, the trends in their accumulation across plant organs and seedlings could not be reliably established. Lin_AT4G07390, the linear counterpart of one of the most abundant circRNA (circ_AT4G07390), had the highest expression among the selected linear transcripts (**Figure 7**). Moreover, the expression level of lin_AT4G07390 increased from seedlings through roots, leaves, and flowers, which resembled the profile observed for circ_AT4G07390. The other two most abundant transcripts were, however, lin_AT3G10260 and lin_AT2G34460, the linear counterparts of circ_AT3G10260 and circ_AT2G34460, respectively. Notably, circ_AT3G10260 and circ_AT2G34460 were the rarest among the tested circRNAs. Similar discrepancies between the levels of circular and linear RNAs were detected for transcripts from AT5G04090 gene, whereas circ_AT5G04090 was one of the most abundant circRNAs, and its linear counterpart lin_AT5G04090 reached only medium expression values, comparable to lin_AT2G48100 and lin_AT1G80750 (the circular isoforms of which, circ_AT2G48100 and circ_AT1G80750, respectively, accumulated to very low levels). Taken together, these data indicated that the abundance of circRNAs was not a simple consequence of the levels of parent gene expression (Pearson correlation coefficient: 0.14). Moreover, we identified 2 circRNAs, circ_AT5G04090 and circ_AT2G39810, that accumulated to considerable levels in all types of samples, and the profile of their accumulation was not correlated with the profile of linear transcript expression. These circRNAs were increased in leaves and flowers, and thus, we considered them organ-specific.

## Discussion

RNA-seq-based identification and quantification of circRNAs poses methodological challenges, which are plausible reasons for the substantial discrepancies in the numbers of individual circRNAs identified in *A. thaliana* in different studies (Wang et al., 2014; Ye et al., 2015). To address this issue, we tested how different experimental procedures and RNA-seq data analysis methods might influence circRNA profiling. First, we compared two commonly used approaches for the preparation of sequencing libraries dedicated to circRNA identification. The first approach (R−) included only rRNA depletion, and the second one (R+) involved additional removal of linear transcripts by a 3′ exonuclease, RNase R. RNase R treatment has been proposed to increase the efficiency of circRNA detection (Jeck et al., 2013) because it enables the enrichment of circRNAs, which otherwise would be missed in RNA-seq due to a large excess of linear transcripts. Our results were consistent with the above opinion. Although in the R+ libraries the average number of reads was approximately half of those in the R− libraries, the number of identified circRNA candidates was three times higher. Thus, the RNase R treatment clearly increased the resolution of the analyses. On the other hand, we found that some circRNAs, which were identified in four biological replicates in R− libraries and were subsequently validated by RT-PCR, were not identified in R+ libraries. This observation can be explained by the plausible nonenzymatic hydrolysis of some circRNAs upon incubation in reaction buffer, which renders them linear and exposes their 3′ ends to RNase R digestion. Nonenzymatic hydrolysis of RNA is a well-known phenomenon that occurs in the presence of multiple factors, including magnesium ions, polymeric organic compounds such as polyvinylpyrrolidone (PVP) and proteins (Bibillo et al., 1999; Bibillo et al., 2000). The hydrolysis leaves a 2’,3’-cyclic phosphate at the 5’ end of the cleavage side and a 5’-hydroxyl group at the 3’ end (Kierzek, 1992). Because RNase R can accommodate 3′-phosphate-terminated substrates (Cheng and Deutscher, 2002), the products of nonenzymatic RNA hydrolysis can be digested by this enzyme. Thus, detailed biochemical studies of the factors that affect the stability of circRNAs upon incubation with RNAse R are required to better understand to what extent reaction conditions impact the composition of circRNA pool. Taken together, RNase R treatment enriches circRNAs, however this procedure may also lead to the loss of some circRNAs. Consequently, we decided that the R− approach was more suitable for the identification and quantification of abundant circRNAs. In addition, regardless of the library type, we observed poor reproducibility of the circRNAs across biological replicates (ranging from 1.3 to 5.0%). The results suggested that the pool of circRNAs contained a substantial fraction of stochastically generated molecules, the physiological relevance of which is likely limited.

Our analyses revealed that the use of RNA-seq data for comparisons of different plant organs requires special attention for several reasons. Obviously, an indispensable prerequisite in such comparative studies is the normalization of RNA-seq data. In our study, the commonly used normalization by the library size was found to be inadequate because the obtained data poorly correlated with the ddPCR results (Pearson correlation coefficient: 0.67). The plausible reason for this result is the natural variation of the transcriptome composition of different organs. Consequently, the applied experimental procedures have different efficiencies, depending on the type of plant material. This phenomenon was evidenced by the varying effectiveness of rRNA depletion from particular organs. Moreover, the organ-specific differences may influence the RNA-seq ability to identify and quantify transcripts, in particular, the less abundant ones, such as circRNAs. To optimize the comparative analyses of circRNAs, we tested five other normalization methods. Four of these methods, namely, normalization by the (i) number of back-spliced reads, (ii) DESeq2 size factor (iii) library size without rRNA and chloroplast RNA reads and (iv) housekeeping gene *ACT2*, reached a Pearson correlation coefficient between the ddPCR and RNA-seq results of 0.76 to 0.77. In the case of single-organ quantitative analyses of circRNA, the correlation was even higher, reaching 0.92. This observation has two implications. First, it supports the opinion that the transcriptome composition has a considerable impact on RNA-seq-based profiling of rare RNA species. Second, this result demonstrates the high reliability of RNA-seq-based quantitative characterization of circRNAs within a particular organ, thus corroborating the results from some previous studies that were not validated by qPCR (Ye et al., 2015; Dou et al., 2017). Moreover, this finding opens a perspective for large-scale single-organ quantitative studies of circRNAs under various conditions and comparisons of their accumulation levels in response to different stimuli.

Our analysis of circRNAs across *A. thaliana* seedlings and organs was based on 127 circRNAs identified by CIRI2 and find_circ programs and supported by at least 10 normalized RNA-seq reads, which corresponded to 50 copies per microgram of total RNA. Based on the provided data, we believe that this set well represents reproducibly generated circRNAs that are most likely to be physiologically relevant. In general, circRNAs were accumulated in an organ-specific manner; however, approximately 41% were ubiquitous. GO analysis revealed that genes giving rise to circRNAs were significantly enriched in those involved in photosynthesis in seedlings, leaves and flowers. However, roots had the highest number of unique circRNAs and most circRNAs of significantly increased accumulation (p-value ≤ 0.05) in comparison to other organs/seedlings. The smallest number of circRNAs with increased accumulation was found for the seedlings. Moreover, our results clearly demonstrated that there was no direct link between the accumulation of circRNAs and their linear counterparts. While the levels of some circRNAs increased with the increase in their linear counterpart transcripts (circ_AT4G07390 and lin_AT4G07390), there was an overall lack of correlation between the accumulation of these two transcriptome fractions (Pearson correlation coefficient: 0.14). This finding indicated that there have to exist specific mechanisms regulating the formation of reproducibly generated circRNAs. What is more, some circRNAs (for example, circ_AT5G04090 and circ_AT2G39810) were selectively increased in particular organs, while the levels of their linear counterparts remained relatively stable throughout the plant. It is tempting to speculate that their biogenesis involves an organ-specific molecular switch that changes the regular splicing pattern. These circRNAs also emerge as attractive candidates for functional studies.

Recently, chloroplasts were described as circRNA production hotspots, and circRNAs were proposed to be involved in photosynthesis (Sun et al., 2016). Our data revealed that back-spliced reads supporting circRNAs derived from the chloroplast genome were indeed prevalent in leaves. These findings raise intriguing questions about how such substantially different processes as nuclear and organellar splicing, could both lead to the formation of circRNAs. However, this issue is complicated by the observation that some organellar mRNAs are products of trans-splicing, in which the exons of two primary transcripts are joined (de Longevialle et al., 2007; Aryamanesh et al., 2017). Some reads derived from such mRNAs can be misclassified as back-spliced reads. In addition, chloroplast group II introns can be excised as full-length circles instead of lariats (Lasda and Parker, 2014). In the above context, chloroplast circRNAs identified by RNA-seq require extensive validation.

Here, we integrated RNA-seq and ddPCR to study *A. thaliana* circRNAs. By using ddPCR results as feedback to improve RNA-seq normalization, we developed a reliable strategy of circRNA comparative analyses. This strategy allowed us to comprehensively characterize circRNAs across plant organs and in seedlings. Our results stimulate further research of organ-specific circRNAs and of the mechanisms that guide a switch between the formation of circular and their linear counterpart transcripts.

## Data Availability Statement

The original contributions presented in the study are publicly available. This data can be found here: https://www.ncbi.nlm.nih.gov/bioproject/PRJNA525820.

## Author Contributions

MF conceived the overall idea of the study. AP, PJ, and MF conceived and designed the experiments. KN performed all bioinformatics analyses. AP, PJ and MF analyzed and discussed the results, MS cultivated plants, isolated RNA and performed PCR/ddPCR. MS and PJ cloned and sequenced PCR products. MS and JP prepared RNA samples for sequencing. JP and LH generated the RNA-seq libraries. AP, KN, MS, and PJ drafted the manuscript. MF was responsible for the final version of the manuscript. All authors contributed to the article and approved the submitted version.

## Funding

This work was financed by the National Science Centre grant number UMO-2014/15/D/NZ2/02305 to AP. Partial financial support was also provided by the Polish Ministry of Science and Higher Education under the KNOW program. The funders had no role in study design, data collection and interpretation, or the decision to submit the work for publication.

## Conflict of Interest

The authors declare that the research was conducted in the absence of any commercial or financial relationships that could be construed as a potential conflict of interest.
