# Supplementary material for: Expression Landscape of circRNAs in Arabidopsis thaliana Seedlings and Adult Tissues
Source: Front Plant Sci. 2020 Sep 10;11:576581. doi: 10.3389/fpls.2020.576581 (PMC7511659; doi:10.3389/fpls.2020.576581)
Supplement: Supplementary file 1 [file DataSheet_1.pdf]

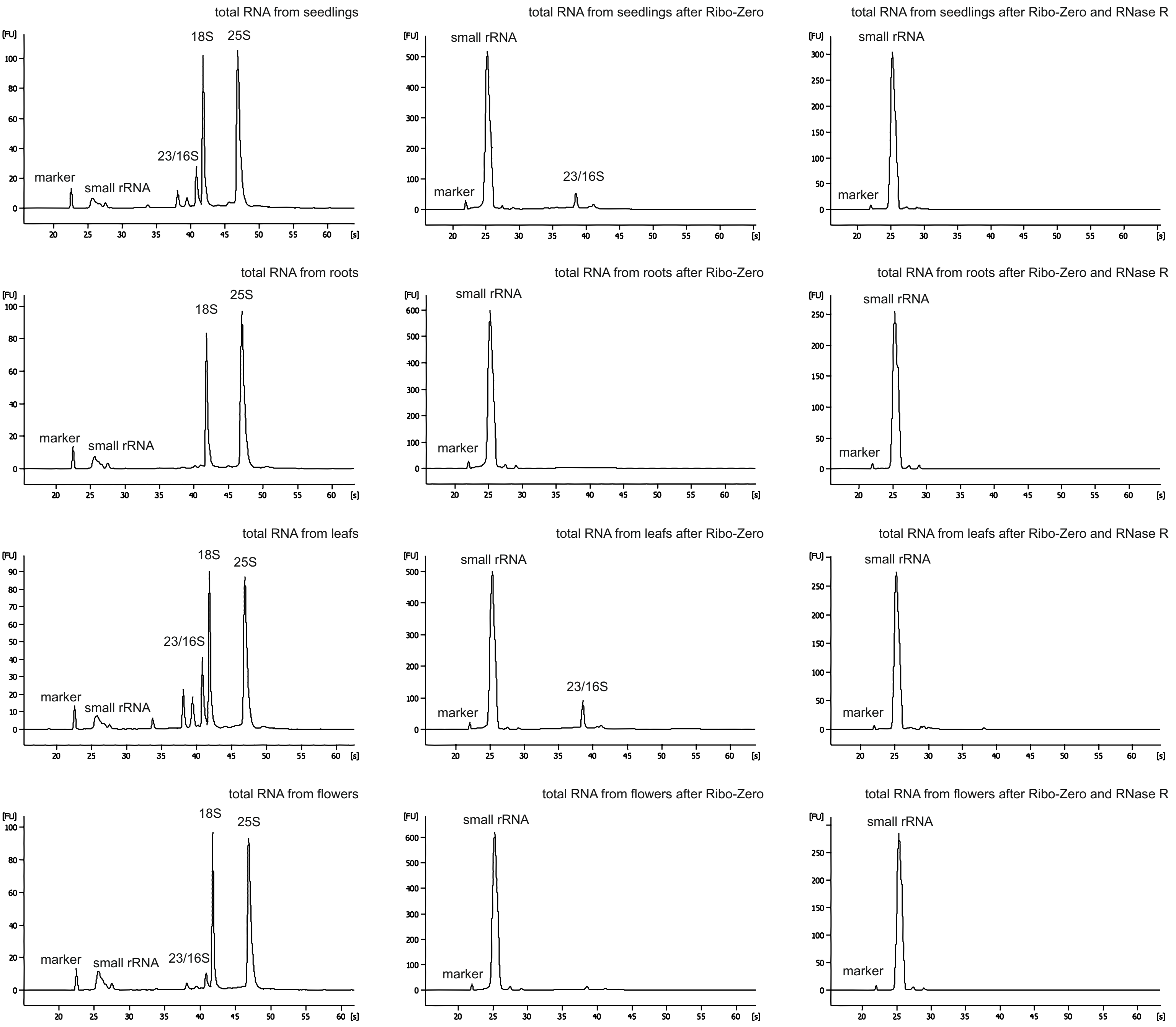

Supplementary Figure 1.
The efficiency of Ribo-Zero and RNase R treatment of total RNA from seedlings and plant organs.

Representative electropherograms (Bioanalyzer, Agilent) of total RNAs (left side), total RNAs after Ribo-Zero treatment (middle), and total RNAs after Ribo-Zero and RNase R treatment (right side) from seedlings, roots, leaves, and flowers. One hundred nanograms of DNase-treated total RNA of each sample was analyzed on the Bioanalyzer, Agilent with RNA Nano Chip in accordance with the manufacturer's instructions. After Ribo-Zero treatment, 3 ng of each sample was analyzed on the Bioanalyzer, Agilent with RNA Pico Chip in accordance with the manufacturer's instructions. After Ribo-Zero and RNase R treatment, 3 ng of each sample was analyzed on the Bioanalyzer, Agilent with RNA Pico Chip in accordance with the manufacturer's instructions. The x-axis represents the resolving time in seconds (s), and the y-axis represents fluorescence (fu). In addition to the cytoplasmic 25S and 18S rRNA peaks, other peaks corresponding to 23S and 16S rRNA from chloroplasts and small rRNAs are present.

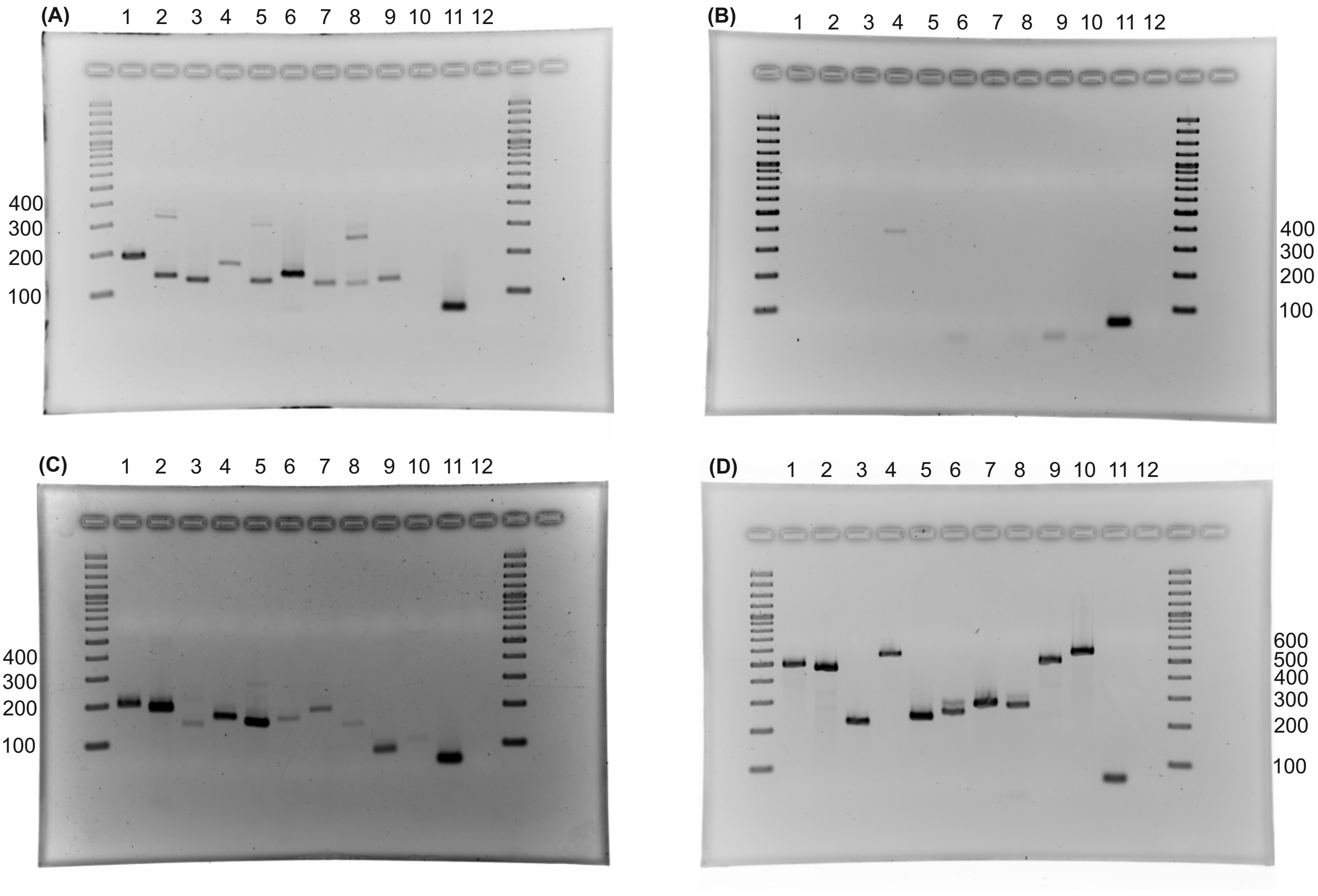

Supplementary Figure 2.
Uncropped gel images used in Figure 1 in the main text.

(A) Uncropped gel image used in panel B, Figure 1.
(B) Uncropped gel image used in panel C, Figure 1.
(C) Uncropped gel image used in panel D, Figure 1.
(D) Uncropped gel image used in panel E, Figure 1.

(A)

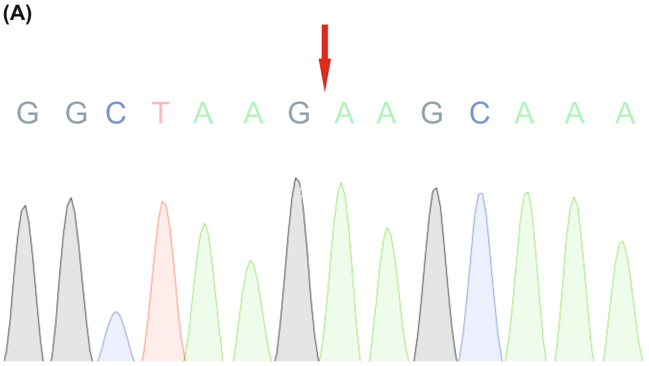

(B)

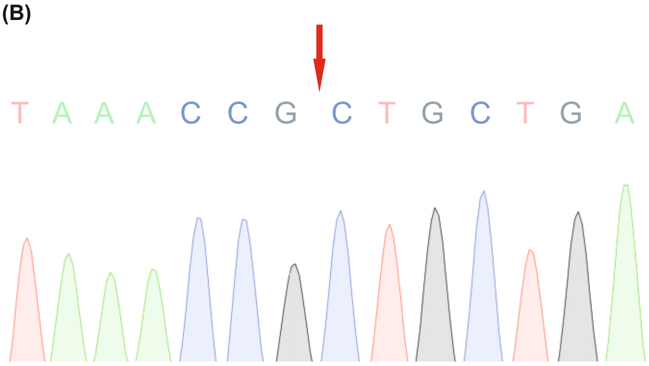

(C)

CCGGCAAGAAGAAGAAGAAAGGCGTAAAAGCCGCTTTTGGTTTGCCTTGGTCAGCAAGGTACAAGGTAGCGTT
AGGTATAGCAGATGCCATAGCTTATTTGCATAATGGAACAGAGCAATGTGTTGTTCACAGAGATATCAAACCCTC
CAATATTCTTCTTTCCTCAAAGAAAATACCAAAGGTAAAGATTCGCGAAACGAATGATGTATTCGATAATTATCGGT
AAGCAAACCAATGATTTGTTTTGACCTGTTCTTTTTCAGTTGTGTGATTTTGGGTTAGCTACTTGGACCGCTGCA
CCTTCGGTTCCTTTCCTCTGCAAGACCGTGAAAGGCACATTCGGGTATTGCTCGAAACTTGCCTTGAGTTGTTT
CTTCTTCTTCTTGCCGGA

Supplementary Figure 3.
Results of Sanger sequencing of PCR products.

(A) Sequencing chromatogram of the circRNA 4 (1:30349032-30349237) amplification product (Figure 1, panel B, lane 4). The red arrow indicates the back-splice site.

(B) Sequencing chromatogram of the circRNA 7 (3:3172073-3172253) amplification product (Figure 1, panel B, lane 7). The red arrow indicates the back-splice site.

(C) The sequence of the mispriming amplification product (Figure 1, panel C, lane 4). It was identified as a fragment of the AT5G37790 locus.

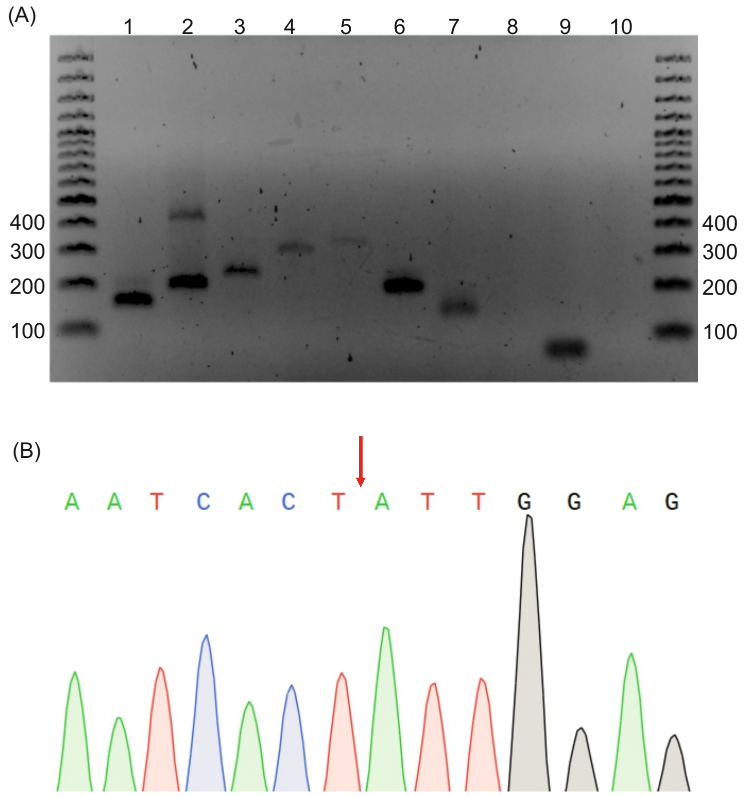

Supplementary Figure 4.

(A) Detection of circRNAs not identified in the R+ libraries. RT-PCR with cDNA from the leaf as template was performed as described in the main text. For PCRs 1-8, divergent (circular specific) primers were used. Lanes 1 to 6 show PCR products corresponding to circRNA candidates that were not identified in the R+ libraries. Lane 7 shows a circRNA derived from the chloroplast chromosome that was found in both libraries. Lane 8 shows a circRNA that was not identified in the R- library in leaves but was detected in seedlings. Lanes 9 and 10 represent positive (leaf cDNA template) and negative (no template) controls, respectively, with convergent primers for ACT2.

CircRNA IDs: 1) 1:20310304-20310540; 2) 4:2719063-2719462; 3) 1:7085326-7088687; 4) 1:26715933-26716400; 5) 1:20066441-20074643; 6) 1:22359475-22363105; 7) Pt:419-556; 8) 3:4922906-4925556.

(B) Sequencing chromatogram of the circRNA 6 (1:22359475-22363105) amplification product (panel A, lane 6). The red arrow indicates the back-splice site.

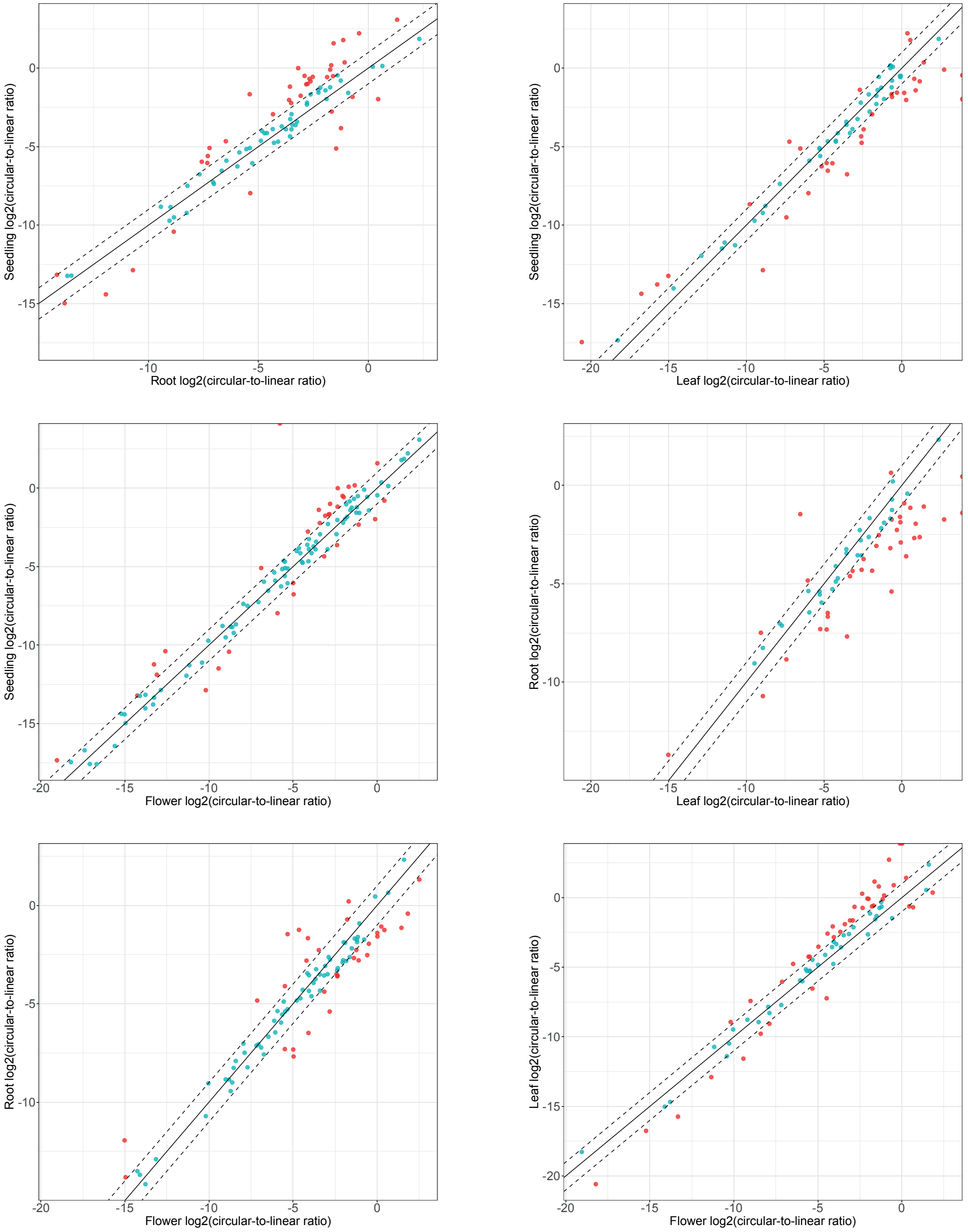

Supplementary Figure 5.

The organs/seedling pairwise comparison of circular to linear ratios. Dashed line: 2-fold cut-off.
